# Cardiac arrhythmias during and after thoracic irradiation for malignancies

**DOI:** 10.1186/s40959-024-00277-3

**Published:** 2024-11-14

**Authors:** Markus B. Heckmann, Jan P. Münster, Daniel Finke, Hauke Hund, Fabian Schunn, Jürgen Debus, Christine Mages, Norbert Frey, Ann-Kathrin Rahm, Lorenz H. Lehmann

**Affiliations:** 1 Department for Cardiology, Angiology, and Pneumology, Heidelberg University Hospital, Heidelberg, Germany; 2 German Centre for Cardiovascular Research: DZHK, Partner Site Heidelberg, Mannheim, Germany; 3 Center for Cardiovascular and Preventive Medicine, ATOS Klinik, Heidelberg, Germany; 4 Department of Radiation Oncology, University Hospital Heidelberg, Heidelberg, Germany; 5 Heidelberg Center for Heart Rhythm Disorders (HCR), University Hospital Heidelberg, Heidelberg, Germany; 6 German Cancer Research Center (DKFZ), Heidelberg, Germany

**Keywords:** Cardiac arrhythmia, Thoracic radiation, Radiation-induced heart disease, Cardiac implantable electrical devices, Radiotherapy, Cardiovascular toxicity

## Abstract

**Background:**

Cardiac arrhythmia has been reported as a significant complication of thoracic radiotherapy. Both bradyarrhythmias and tachyarrhythmias have been reported, highlighting the arrhythmia-modulating potential of radiation in certain oncologic therapies. This study aimed to analyse the arrhythmic burden in patients with cardiac implantable electrical devices (CIEDs) undergoing thoracic irradiation, examining both immediate effects of radiotherapy and long-term sequelae post-therapy.

**Methods and results:**

A retrospective cohort study was conducted involving patients with CIEDs who received thoracic radiotherapy between January 2012 and December 2022. Two distinct analyses were performed involving (1) daily CIED follow-ups during radiotherapy and (2) long-term arrhythmic outcomes post-therapy. For long-term outcomes, Patients were matched in a 1:2 ratio with non-irradiated controls based on age, sex, cardiovascular risk factors, cardiac disease, and beta-blocker use. Statistical analyses included negative binomial regression and propensity score matching. A total of 186 patients underwent daily CIED monitoring during radiotherapy, with 79 receiving thoracic irradiation. Thoracic irradiation was negatively associated with atrial arrhythmia (OR 0.11 [0.02;0.70, 95% CI], adjusted *p* = 0.0498) and there was a tendency towards less ventricular events (OR 0.14 [0.02;1.41, 95% CI], adjusted *p* = 0.3572) during radiotherapy in a univariate regression analysis. This association was not significant in the multivariate (OR 0.44 [0.10;1.80, 95%-CI], *p* = 0.16) model including a history of atrial fibrillation, diabetes and beta-blocker use. Coronary artery disease was associated with an increase in atrial and ventricular arrhythmia. For the long-term analysis, 122 patients were followed up after thoracic (*N* = 33) and non-thoracic radiation (*N* = 89) and compared to 244 matched controls drawn from approximately 10.000 CIED-patients. There was no significant increase in arrhythmic events compared to controls over a median follow-up of 6.6 months. A previous history of ventricular or atrial arrhythmic events was the strongest predictor for events during the follow-up.

**Conclusion:**

Thoracic radiotherapy can be safely administered in patients with CIEDs. However, patients with a history of arrhythmia are more prone to arrhythmic events during and after radiation. These findings highlight the need for personalized arrhythmia management strategies and further research to understand the mechanisms underlying the antiarrhythmic effects of thoracic radiation.

**Graphical Abstract:**

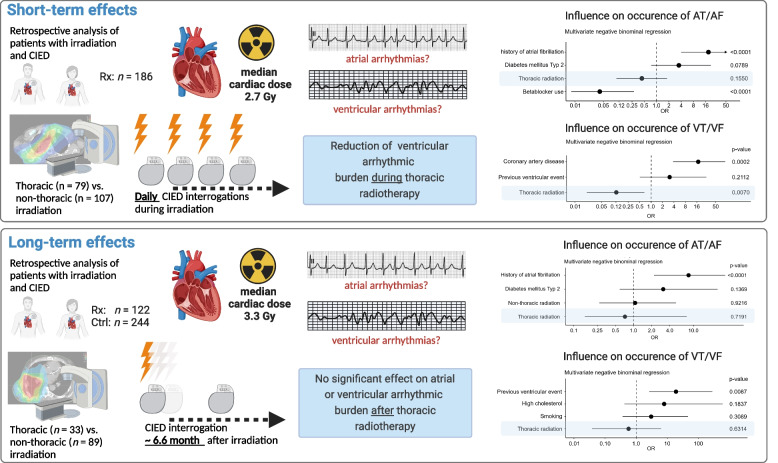

**Supplementary Information:**

The online version contains supplementary material available at 10.1186/s40959-024-00277-3.

## Introduction

Cardiovascular disease and cancer are the two leading causes of death worldwide [1]. In Germany, half of all deaths can be attributed to either category [2]. While the number of people suffering from oncological diseases continues to grow, the mortality rate for many oncological diseases has decreased over the past decade [1]. This is leading to an increasing number of cancer survivors further stressing the importance of reducing cardiovascular toxicity of these therapies [3]. Approximately 35% of all oncology patients receive radiation therapy within the first year of diagnosis, and half of all cancer patients will receive radiation therapy at some point during their disease [4, 5]. If the heart is in or close to the radiation field, radiation induced heart disease (RIHD) may occur [6].

RIHD encompasses a range of clinical manifestations, including pericardial, valvular, and coronary artery disease, with cardiac arrhythmias emerging as a significant concern following thoracic radiotherapy [7]. Both bradyarrhythmias and tachyarrhythmias have been documented, illustrating the arrhythmogenic potential of radiation and certain oncologic therapeutics [8–11]. Historical reports and more contemporary studies have detailed the range of conduction abnormalities and arrhythmias associated with radiation, from nonspecific ECG changes to severe atrioventricular blocks and tachyarrhythmias [12–15].

Recent studies have also explored the therapeutic potential of radiation in the treatment of refractory arrhythmias, suggesting that targeted radiation may have rhythm stabilizing effects [10, 16–19]. Preliminary results from these interventions suggest that stereotactic radiation, when precisely delivered to arrhythmogenic foci, can significantly reduce arrhythmic burden, a promising finding that could help the treatment of certain severe cases. However, the biological basis of this effect, which may involve changes in the expression of key ion channels and gap junction proteins, remains to be fully elucidated [20, 21].

At first glance these recent findings might seem ad odds with the more historic findings on radiation induced arrhythmic events. Studies on the incidence of arrhythmias after thoracic radiotherapy are challenging due to the lack of standardized follow-up protocols and only intermittent ECG monitoring [11]. Patients with cardiac implantable electrical devices (CIED), who represent up to 1% of the patient population, may fill the gap in this area as they are under continuous rhythmic monitoring [22]. The aim of this study was to analyse the arrhythmic ventricular and supraventricular load in CIED patients undergoing thoracic irradiation.

## Methods

In a mono-centric retrospective cohort study we investigated the immediate and long-term impact of radiotherapy on cardiac conduction and arrhythmic events in patients with an CIED. It was conducted at the University Hospital in accordance with the principles of the Declaration of Helsinki. Approval was granted by the ethics committee of the University Hospital of Heidelberg (S-240/2017). The analysis consisted of two parts. Daily CIED follow-ups on the same day after patients received radiotherapy, as well as a cohort of CIED patients followed over time after completion of radiotherapy.

### Study population and design

To understand the immediate cardiac arrhythmogenic effects of radiotherapy (1), all consecutive patients, who underwent daily CIED follow-up exams directly after each session of radiotherapy between January 2012 and December 2022 in the Department of Cardiology, Angiology, and Pneumology at Heidelberg University Hospital were included. Patient characteristics and results of the exams were retrieved from the hospital information system using natural language processing and pattern matching as described below.

**Fig. 1:**
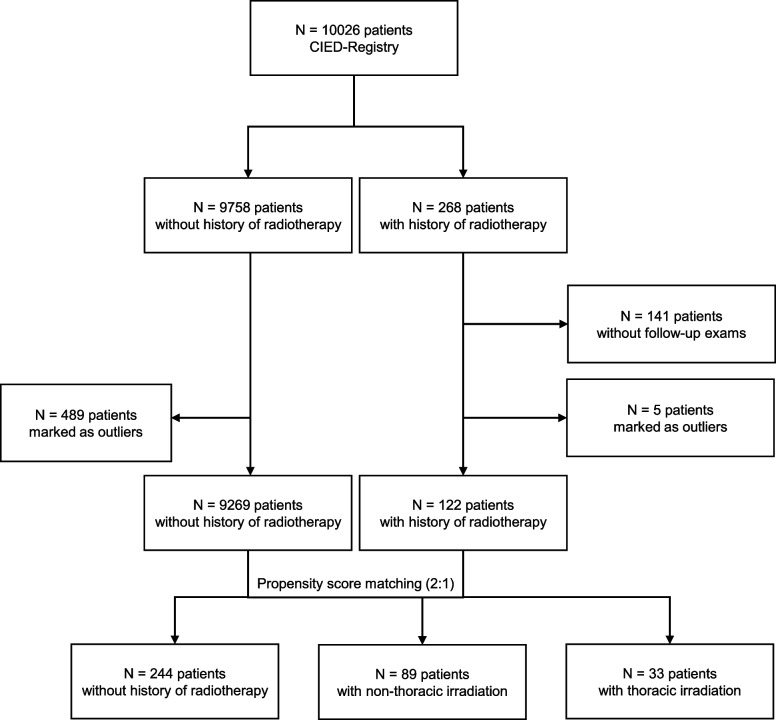
Flowchart of patient selection and categorization. A total of 10,026 patients were identified from the CIED-Registry. Two hundred fifty-six of these patients also had radiotherapy. One hundred twenty-two patients had a history of radiotherapy with a follow-up exam after radiotherapy. After propensity score matching (2:1), the cohort included 122 patients with a history of radiotherapy and 244 patients without. Thirty-three patients received thoracic irradiation and 89 patients had non-thoracic irradiation

To improve our understanding of long-term cardiac rhythmogenic effects from radiation in a previously diseased patient population (2), we analysed the data from all consecutive patients undergoing routine CIED scans between January 2012 and December 2022 in the at the Department of Cardiology, Angiology, and Pneumology at Heidelberg University Hospital. All pertinent medical information including current medical treatment, previous diagnoses, and data from the CIED scans were extracted either directly from the system or, if necessary, using natural language processing and pattern matching as described below. CIED-scans with unplausible or extreme values with a deviation greater three times the standard deviation from the mean were excluded from the analysis. Patients whose CIEDs were scanned after a course of radiotherapy were used as the interventional cohort and matched non-irradiated patients were used as controls. Matching was performed with the *MatchIt* package [23] in a 1:2 ratio (irradiated/non-irradiated). Age, sex, cardiovascular risk factors (arterial hypertension, dyslipoproteinemia, smoking, diabetes, family history, obesity), cardiac diseases (dilated cardiomyopathy, hypertrophic cardiomyopathy, coronary artery disease, atrial fibrillation, atrioventricular block, sick sinus syndrome), and beta-blocker use at baseline were used as matching variables. Propensity scores were calculated using logistic regression. Pairwise matching was then performed applying the nearest neighbour method. A flow chart is shown in Fig. 1.

### Data preparation

Data extraction and processing were performed using R (version 4.3.1). The *dplyr* (version 1.1.2) and *tidyr* (version 1.3.0) packages were used for data wrangling and *stringr* (version 1.5.0) for full-text extraction using pattern matching. Medical therapy including betablocker therapy and cardiovascular risk factors such as arterial hypertension, dyslipoproteinemia, smoking status, diabetes mellitus, positive family history for cardiovascular events, known coronary heart disease, known cardiomyopathies, known atrial fibrillation, cardiac conduction diseases such as sick-sinus-syndrome or atrioventricular blocks, percentage of atrial pacing, percentage of ventricular pacing, mode switch time, episodes of atrial tachycardia or atrial fibrillation (AT/AF), non-sustained ventricular tachycardia (nsVT), sustained ventricular tachycardia (VT), and events of ventricular fibrillation (VF) were extracted. After each extraction a representative sample was used to manually check for extraction errors. Full-text extraction functions were optimised until error rates were below a preset threshold of 5%. In addition to automated full-text extraction, all documents pertaining to the immediate follow-up CIED scans directly after radiotherapy were checked manually. To assess the immediate effects of irradiation, the number of atrial (AT/AF) and ventricular (nsVT, VT, VF) episodes occurring during radiotherapy was summed for each patient. For long-term analysis, the mean number of atrial (AT/AF) and ventricular (nsVT, VT, VF) episodes occurring during follow-up was calculated per patient.

### Statistical analysis

Continuous variables were presented as median and interquartile range. Categorical variables were presented as frequency counts and percentages. For initial data visualisation and analysis, Kernel density plots were created using the *ggpubr* package (version 0.6.0). Patient characteristics were summarized in tables using the *tableone* package (version 0.13.2). Potential differences in categorical variables were tested for independence using the chi-squared test or fisher´s exact test. Continuous variables were first tested for normal-distribution using the Shapiro–Wilk test. Further analysis was performed using the non-parametric Kruskal–Wallis test. The eta-squared (η^2^) was calculated as a measure of the variance explained by the predictor. In general, two-sided tests were performed. A *P-*value less than 0.05 was considered significant. In case of significant group differences between three cohorts, further post-hoc tests were performed. The Kolmogorov–Smirnov test was used to test for equality of distributions. Pairwise Wilcoxon tests applying the holm-bonferroni method for p level adjusted were used. For better comparability, the η-squared (η^2^) was also used as a measure of variance clearance, which is equivalent to the coefficient of determination R^2^. Calculations were performed using the *sjstats* package (version 0.18.2).

Univariate negative binomial regression models were fitted to the data. The models were calculated using the *MASS* package (version 7.3–60.0.1). Predictors for the multivariate models were selected using Akaike’s information criterion. Finally, the odds ratio and 95% confidence interval (CI) were calculated as a measure of the effect size of each predictor. The calculated ORs, confidence intervals, and corresponding *P-*values of the variables were used to present the results. Correlations between mean heart dose and the sum of arrhythmic episodes were evaluated using Kendall’s Tau coefficient, providing insight into the relationship between radiation dose and arrhythmia burden.

## Results

### Datasets and quality control

To determine full-text data extraction quality a random data was manually extracted from a random sample of 500 patients. The accuracy of the automated full-text extraction was above 95% for all data entry points (see Supplementary Table 1).

### Direct arrhythmic effects of thoracic radiation

#### Patient characteristics

A total of 186 CIED patients were scanned daily for the duration of radiotherapy, of which 79 (42%) patients were irradiated to the chest. Patients who received thoracic radiotherapy had a median age of 76.3 years (67.6–79.6 IQR) years and 78.5% were male. Patients receiving non-thoracic radiotherapy had a median age of 75.6 years (69.5–80.2 IQR) years and 76.6% were male. Patients receiving non-thoracic irradiation received a higher total dose than patients receiving thoracic irradiation (60.0 Gy [40–72 IQR] vs. 49.0 Gy [31–59 IQR], *p* < 0.001). In addition, more patients with thoracic radiation received concurrent chemotherapy during radiotherapy than patients receiving (non-)thoracic irradiation (31.6% vs. 18.7%, *p* = 0.05). The median cardiac dose in the thoracic irradiated patients was 2.7 Gy (0.6–8.1 IQR). Cardiovascular risk factors, including arterial hypertension, dyslipoproteinemia, smoking, diabetes, and coronary heart disease, were comparably distributed between the two groups. A complete list of patient characteristics is provided in Table 1.

**Table 1:** Patient characteristics. Arrhythmia during radiotherapy. FH: Family history of, CvD: Cardiovascular disease. CRT: Cardiac resynchronisation therapy, ICD: Implantable cardioverter/defibrillator. Count data is reported as counts and its respective percentage. Continuous data is reported with its median and IQR

	**Thoracic irradiation (*N* = 79)**	**Non-thoracic irradiated** **(*N* = 107)**	***P-*value**
**Age (years)**	76.3 [67.65–74.20]	75.6 [69.45–80.15]	0.90
**Sex**			
Female	17 (21.5%)	25 (23.4%)	0.87
Male	62 (78.5%)	82 (76.6%)	
**Arterial hypertension**	33 (41.8%)	47 (43.9%)	0.88
**Dyslipoproteinemia**	19 (24.1%)	26 (24.3%)	1.00
**Smoking**	10 (12.7%)	10 (9.3%)	0.50
**Diabetes**	17 (21.5%)	20 (18.7%)	0.71
**Positive family history for CvD**	4 (5.1%)	4 (3.7%)	0.72
**Obesity**	7 (8.9%)	5 (4.7%)	0.37
**Coronary disease**	33 (41.8%)	50 (46.7%)	0.56
**Dilated cardiomyopathy**	3 (3.8%)	10 (9.3%)	0.17
**Hypertrophic cardiomyopathy**	1 (1.3%)	3 (2.8%)	0.65
**Atrial fibrillation**	27 (34.2%)	41 (38.3%)	0.67
**Sick sinus syndrome**	12 (15.2%)	19 (17.8%)	0.68
**AV block (all grades)**	19 (24.1%)	27 (25.2%)	0.87
**Beta-blocker therapy**	17 (21.5%)	17 (15.9%)	0.34
**Device type**			
CRT	7 (8.9%)	6 (5.6%)	0.52
Pacemaker	50 (63.3%)	76 (71.0%)	
ICD	22 (27.8%)	25 (23.4%)	
**concomitant chemotherapy**	25 (31.6%)	20 (18.7%)	0.05
**Total dose (Gy)** Median [IQR]	49.0 [31–59]	60.0 [40–72]	< 0.001
**Oncologic disease**			
None	0 (0%)	3 (2.8%)	< 0.001
Others	17 (21.5%)	55 (51.4%)	
Lung cancer	39 (49.4%)	1 (0.9%)	
Glioblastoma	0 (0%)	9 (8.4%)	
Colorectal cancer	1 (1.3%)	4 (3.7%)	
Breast cancer	9 (11.4%)	0 (0%)	
Oesophageal cancer	12 (15.2%)	0 (0%)	
Prostate cancer	1 (1.3%)	35 (32.7%)	
**Occurrence of episodes (AT/AF)**			
Yes	14 (17.7%)	12 (11.2%)	0.28
No	65 (82.3%)	95 (88.8%)	
**Occurrence of episodes (ventricular)**			
Yes	5 (6.3%)	12 (11.2%)	0.31
No	74 (93.7%)	95 (88.8%)	
**HFrEF at baseline**			
Yes	19 (24.1%)	25 (23.4%)	0.32
No	8 (10.1%)	19 (17.8%)	
Unknown	52 (65.8%)	63 (58.9%)	
**Antiarrhythmic therapy**			
Amiodarone	1 (1.3%)	1 (0.9%)	1
None	78 (98.7%)	106 (99.1%)	

#### Atrial arrhythmic burden under radiotherapy

**Fig. 2:**
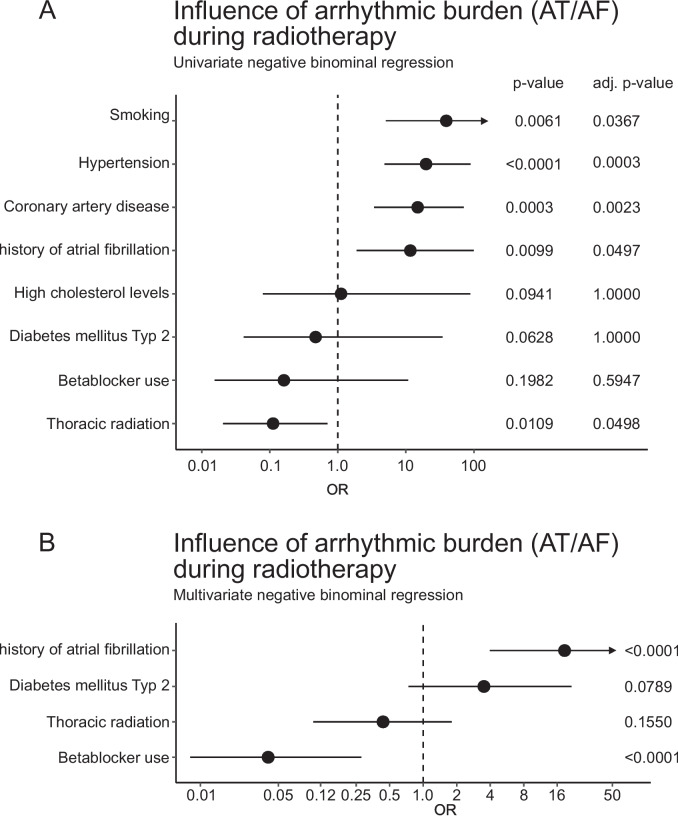
Atrial arrhythmic burden during radiotherapy. **A** Univariate negative binomial regression analysis assessing the impact of include smoking, hypertension, coronary artery disease, history of atrial fibrillation, high cholesterol levels, diabetes mellitus type 2, beta-blocker use, and thoracic radiation on arrhythmic burden. **B** Multivariate negative binomial regression analysis on history of atrial fibrillation, diabetes mellitus type 2, thoracic radiation, and beta-blocker use. Odds ratios (OR) and confidence intervals (CI) are displayed along with *P-*values and adjusted *P-*values (holm-bonferroni)

In the cohort undergoing thoracic radiotherapy, 14 patients (17.7%) experienced atrial arrhythmia, while in the non-thoracic radiotherapy cohort, 12 patients (11.2%) experienced atrial arrhythmias (*p* = 0.28). Univariate regression analysis revealed several significant factors influencing the arrhythmic burden during radiotherapy. Smoking (OR 39.24 [5.09;2228.14, 95%-CI], adjusted *p* = 0.0367), known hypertension (OR 19.83 [4.85;88.97, 95%-CI], adjusted *p* = 0.0003), known coronary artery disease (OR 14.92 [3.41;70.62, 95%-CI], adjusted *p* = 0.0023) and a history of atrial fibrillation (OR 11.64 [1.89;99.61, 95%-CI], adjusted *p* = 0.0497) were significantly associated with atrial arrhythmic burden. Thoracic radiation (OR 0.11 [0.02;0.70, 95%-CI], adjusted *p* = 0.0498), on the other hand, was negatively correlated. Other factors such as high cholesterol levels, diabetes mellitus type 2, and beta-blocker use were not statistically significant after adjustment (see Fig. 2).

Multivariate analysis further refined these associations, highlighting the most impactful predictors. A history of atrial fibrillation (OR 18.61 [3.97;93.91, 95%-CI], *p* < 0.0001) remained a strong predictor of increased arrhythmic burden, even when considering other variables. Diabetes mellitus type 2 (OR 3.51 [0.74;21.44, 95%-CI], *p* = 0.0789) and thoracic radiation (OR 0.43 [0.10;1.80, 95%-CI], *p* = 0.1550) showed trends towards significance but did not reach statistical significance in the multivariate context. This might be indicative of an association of thoracic radiation with other predictors. Beta-blocker use (OR 0.04 [0.01;0.28, 95%-CI], *p* < 0.0001) emerged as a significant protective factor (see Fig. 2).

#### Ventricular arrhythmic burden under radiotherapy

**Fig. 3:**
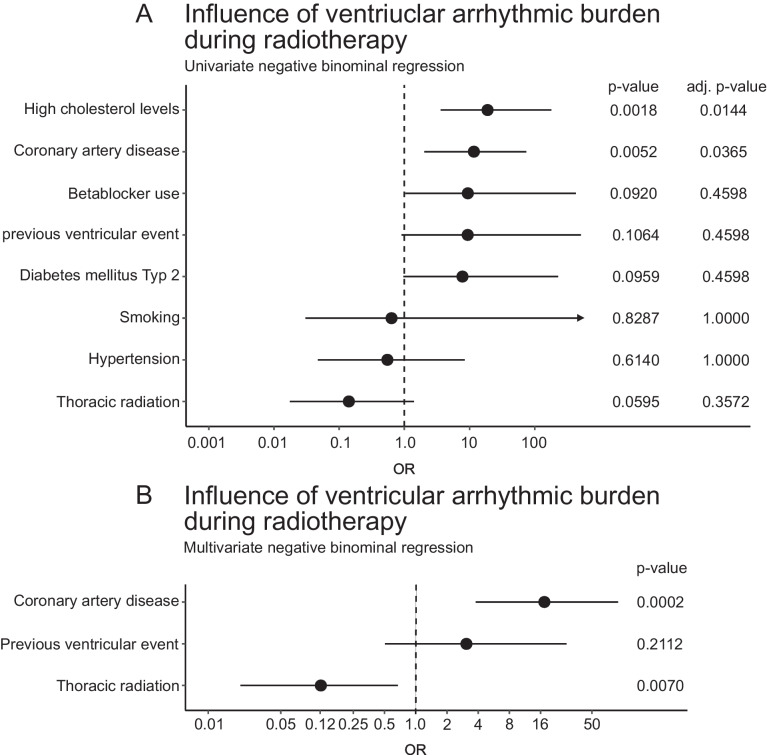
Ventricular arrhythmic burden during radiotherapy. **A** Univariate negative binomial regression analyzing the impact of high cholesterol levels, coronary artery disease, beta-blocker use, previous ventricular event, diabetes mellitus type 2, smoking, hypertension, and thoracic radiation on ventricular arrhythmic burden. **B** Multivariate negative binomial regression analysis focusing on coronary artery disease, previous ventricular event, and thoracic radiation. Odds ratios (OR) and confidence intervals (CI) are displayed along with *P-*values and adjusted *P-*values

In the cohort undergoing thoracic radiotherapy, 5 patients (6.4%) experienced ventricular arrhythmia, while in the non-thoracic radiotherapy cohort, 12 patients (11.2%) experienced ventricular arrhythmias (*p* = 0.31). High cholesterol levels (OR 18.89 [3.61;179.17, 95%-CI], adj. *p* = 0.0144) and coronary artery disease (OR 11.65 [2.03;74.38, 95%-CI], adjusted *p* = 0.0365) were significantly associated with an increased ventricular arrhythmic burden during radiotherapy in a univariate negative binominal regression. Although there were some trends observed for beta-blocker use (OR 9.41 [1.04;425.98, 95%-CI], *p* = 0.0920), previous ventricular event (OR 9.36 [0.91;510.48, 95%-CI], *p* = 0.1064), and diabetes mellitus type 2 (OR 7.78 [0.97;227.42, 95%-CI], *p* = 0.0959), these did not reach statistical significance after adjustment (see Fig. 3).

Multivariate analysis revealed that coronary artery disease (OR 17.38 [3.79;89.23, 95%-CI], *p* = 0.0002) remained a significant predictor of increased ventricular arrhythmic burden, while thoracic radiation (OR 0.12 [0.02;0.67, 95%-CI], *p* = 0.0070) was negatively correlated, reducing the risk of ventricular arrhythmias. Previous ventricular events did not reach statistical significance (OR 3.08 [0.51;28.36, 95%-CI], *p* = 0.2112) in the multivariate context (see Fig. 3).

### Long-term arrhythmic effects of thoracic irradiation

#### Patient characteristics

A total of 10,026 patients were identified from the CIED-Registry. Two hundred fifty-six of these patients also had radiotherapy. One hundred twenty-two patients had a history of radiotherapy with a follow-up exam after radiotherapy. After propensity score matching (2:1), the cohort included 122 patients with a history of radiotherapy and 244 patients without. Thirty-three patients received thoracic irradiation and 89 patients had non-thoracic irradiation. The median cardiac dose in the thoracic irradiated patients was 3.3 Gy (0.9–7.1 IQR). Patient characteristics are summarized in Table 2.

**Table 2:** Patient characteristics. Arrhythmia after radiotherapy. FH: Family history of, CvD: Cardiovascular disease. CRT: Cardiac resynchronisation therapy, ICD: Implantable cardioverter/defibrillator. CMP: Cardiomyopathy. Count data is reported as counts and its respective percentage. Continuous data is reported with its median and IQR

		**Radiotherapy**		***P***-**value**
	**No Radiation** **(*N* = 244)**	**Thoracic (*N*** **= 33)**	**Non-thoracic (*N*** **= 89)**	
**Age (years)**	77.7 [71.20–83.53]	76.8 [69.70–80.70]	75.6 [71.50–80.30]	0.25
**Sex**				
Female	48 (19.7%)	7 (21.2%)	15 (16.9%)	0.79
Male	196 (80.3%)	26 (78.8%)	74 (83.1%)	
**Arterial hypertension**	94 (38.5%)	11 (33.3%)	34 (38.2%)	0.84
**Dyslipoproteinemia**	71 (29.1%)	9 (27.3%)	30 (33.7%)	0.65
**Smoking**	37 (15.2%)	5 (15.2%)	16 (18.0%)	0.82
**Diabetes**	29 (11.9%)	4 (12.1%)	15 (16.9%)	0.54
**Positive FH for CvD**	15 (6.1%)	1 (3.0%)	8 (9.0%)	0.47
**Obesity**	26 (10.7%)	3 (9.1%)	13 (14.6%)	0.55
**Coronary disease**	129 (52.9%)	17 (51.5%)	53 (59.6%)	0.54
**Dilated CMP**	17 (7.0%)	1 (3.0%)	7 (7.9%)	0.66
**Hypertrophic CMP**	10 (4.1%)	0 (0%)	5 (5.6%)	0.47
**Atrial fibrillation**	89 (36.5%)	10 (30.3%)	35 (39.3%)	0.65
**Sick sinus syndrome**	39 (16.0%)	7 (21.2%)	14 (15.7%)	0.77
**AV block (all grades)**	59 (24.2%)	10 (30.3%)	28 (31.5%)	0.36
**Beta-blocker therapy**	36 (14.8%)	7 (21.2%)	12 (13.5%)	0.54
**Device type**				
CRT	19 (7.8%)	1 (3.0%)	7 (7.9%)	0.76
Pacemaker	118 (48.4%)	18 (54.5%)	43 (48.3%)	
ICD	44 (18.0%)	7 (21.2%)	21 (23.6%)	
Not reported	63 (25.8%)	7 (21.2%)	18 (20.2%)	
**Follow-up (days)**	139 [90–725]	160 [114–937]	299 [116–1384]	0.02
**Time since radiation (d)**	NA	321 [21–888]	1130 [546–2274]	< 0.001
**Total dose (GyE)**	NA	54.0 [35–66]	60.0 [30–68]	0.24
**Occurrence of episodes (AT/AF)**				
Yes	56 (23.0%)	13 (39.4%)	25 (28.1%)	0.11
No	188 (77.0%)	20 (60.6%)	64 (71.9%)	
**Occurrence of episodes (ventricular)**				
Yes	53 (21.7%)	7 (21.2%)	25 (28.1%)	0.45
No	191 (78.3%)	26 (78.8%)	64 (71.9%)	
**HFrEF at baseline**				
Yes	67 (27.5%)	8 (24.2%)	26 (29.2%)	0.94
No	57 (23.4%)	6 (18.2%)	24 (27.0%)	
Unknown	120 (49.2%)	19 (57.6%)	39 (43.8%)	
**Antiarrhythmic therapy**				
Amiodarone	3 (1.2%)	1 (3.0%)	1 (1.1%)	0.67
None	241 (98.8%)	32 (97%)	88 (98.9%)	

#### Atrial arrhythmic burden following radiotherapy

**Fig. 4:**
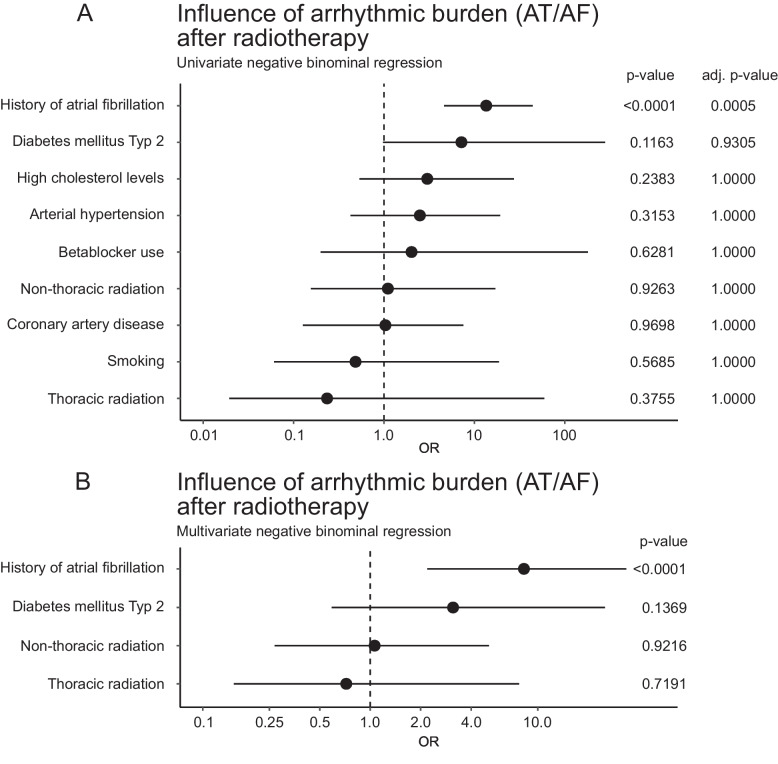
Atrial arrhythmic burden during radiotherapy. **A** Univariate negative binomial regression analysis assessing the impact of history of atrial fibrillation, diabetes mellitus type 2, high cholesterol levels, arterial hypertension, beta-blocker use, non-thoracic radiation, coronary artery disease, smoking, and thoracic radiation. **B** Multivariate negative binomial regression analysis highlighting significant factors including history of atrial fibrillation, diabetes mellitus type 2, non-thoracic radiation, and thoracic radiation. Odds ratios (OR) and confidence intervals (CI) are displayed alongside *P-*values and adjusted *P-*values

During follow up after thoracic or non-thoracic radiotherapy, atrial arrhythmia occurred in 13 patients (39.4%) and 25 patients (28.1%) respectively. In the matched control cohort, atrial arrhythmia was observed in 56 patients (23.0%, *p* = 0.11). Univariate binominal regression analyses showed that a history of atrial fibrillation (OR 13.52 [4.61;44,23, 95%-CI], adjusted *p* = 0.0005) was significantly associated with an increased atrial arrhythmic burden after radiotherapy. Other factors such as diabetes mellitus type 2, high cholesterol levels, arterial hypertension, beta-blocker use, non-thoracic radiation, coronary artery disease, smoking, and thoracic radiation did not show significant associations with arrhythmic burden (see Fig. 4).

In a multivariate analysis a history of atrial fibrillation (OR 8.27 [2.19;33.77, 95%-CI], *p* < 0.0001) remained the only significant predictor of increased arrhythmic burden. Other factors, including diabetes mellitus type 2 (OR 3.12 [0.59;25.18, 95%-CI], *p* = 0.1369), non-thoracic radiation (OR 1.06 [0.27;5.11, 95%-CI], *p* = 0.9216), and thoracic radiation (OR 0.72 [0.15;7.73, 95%-CI], *p* = 0.7191) were not significant (see Fig. 4).

#### Ventricular arrhythmic burden following radiotherapy

**Fig. 5:**
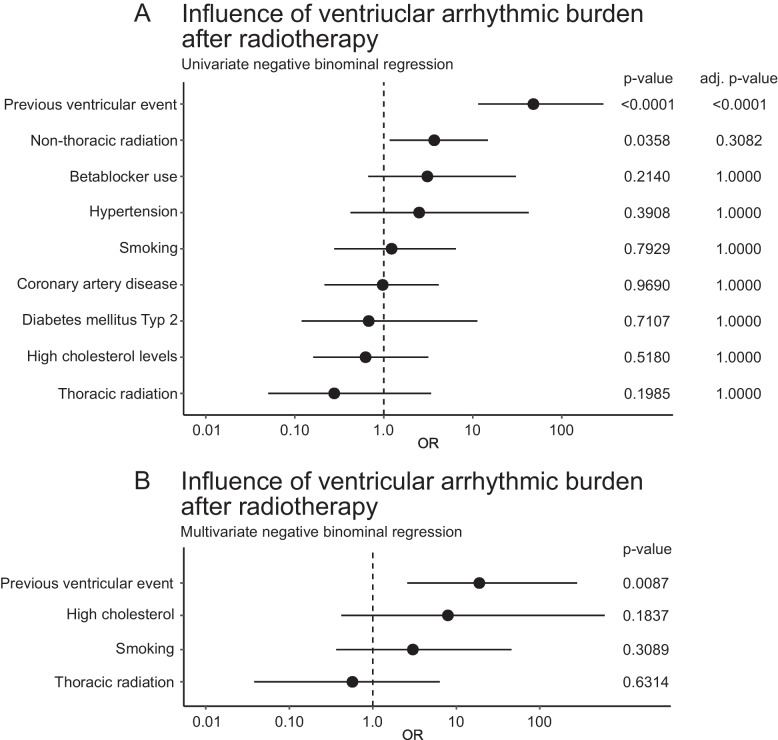
Ventricular arrhythmic burden after radiotherapy. **A** Univariate negative binomial regression analysis assessing the impact of previous ventricular event, non-thoracic radiation, beta-blocker use, hypertension, smoking, coronary artery disease, diabetes mellitus type 2, high cholesterol levels, and thoracic radiation. **B** Multivariate negative binomial regression analysis on ventricular arrhythmic burden including previous ventricular event, high cholesterol levels, smoking, and thoracic radiation. Odds ratios (OR) and confidence intervals (CI) are displayed alongside *P-*values and adjusted *P-*values

During follow up after thoracic or non-thoracic radiotherapy, ventricular arrhythmia occurred in 7 patients (21.2%) and 25 patients (28.1%) respectively. In the matched control cohort, ventricular arrhythmia was observed in 53 patients (21.7%, *p* = 0.45). In a univariate binominal regression analysis, we found previous ventricular events (OR 48 [12;293, 95%-CI], adjusted *p* < 0.0001) to be significantly associated with an increased ventricular arrhythmic burden after radiotherapy. Non-thoracic radiation (OR 3.69 [1.16;14.81, 95%-CI], *p* = 0.0358) was also significantly associated with ventricular events before adjusting for multiple testing. Other factors, including beta-blocker use, hypertension, smoking, coronary artery disease, diabetes mellitus type 2, high cholesterol levels, and thoracic radiation, did not show significant (see Fig. 5).

In the multivariate analysis (Panel B), a previous ventricular event (OR 18.84 [2.59;279.33, 95%-CI], *p* = 0.0087) remained a significant predictor of increased ventricular arrhythmic burden. High cholesterol levels (OR 7.92 [0.42;596.67, 95%-CI], *p* = 0.1837), smoking (OR 3.01 [0.36;45.63, 95%-CI], *p* = 0.3089), and thoracic radiation (OR 0.57 [0.04;6.30, 95%-CI], *p* = 0.6314) did not show a significant association (see Fig. 5).

## Discussion

The current study comprehensively analyses the impact of radiotherapy on atrial and ventricular burden during as well as after radiotherapy using data gathered from patients with cardiac implantable devices undergoing radiotherapy. To our knowledge, our analysis comprises one of the largest cohorts of CIED-patients undergoing radiotherapy. In accordance with previously published data, radiotherapy can be considered safe in these patients when planned and performed in accordance with current recommendations [24–26]. In our study, we further harnessed the possibility to monitor our patients for arrhythmia during and after radiation.

In our analysis, we noted a protective short-term effect of thoracic radiation when compared to non-thoracic radiation for ventricular and atrial arrhythmia. This finding is well in line with previous preliminary studies using cardiac radiation as a bail-out strategy for severe ventricular arrhythmia [20, 21, 27]. However, the median cardiac doses for ablative cardiac irradiation are more than ten times higher and are applied only once. Our cohorts were treated repetitively and with much lower doses. On a molecular level, ablative cardiac radiotherapy temporarily leads to a restoration of the electrical conductivity of the heart by increasing expression of voltage-gated sodium channels and connexin 43 [20, 27]. It is tempting to speculate that these protective molecular effects may also be seen with repetitive lower doses. Further basic research is needed to elucidate the molecular changes after repeated low-dose irradiation of the heart.

From a cardio-oncologic view, radiotherapy is associated with inflammation and fibroblast activation when the myocardium is in the radiation beam [28]. We consequently expected an increase in atrial and ventricular events following radiotherapy due to the expected inflammatory response. Our long-term analysis did not show a significant increase of non-thoracic radiation for ventricular events. Interestingly, thoracic radiation was associated with a decrease in both atrial and ventricular events. The differences in ventricular episodes observed between thoracic and non-thoracic irradiated patients during radiotherapy might also be in part attributed by concomitant therapy. Androgen deprivation therapy, for example, has been associated with QT prolongation and could lead to an increase in torsades de pointes tachycardia [29]. However, most episodes were in fact non sustained ventricular tachycardia.

Several limitations need to be acknowledged. First, the study’s retrospective design may introduce selection bias, and the reliance on existing medical records can lead to incomplete data capture. For example, left ventricular ejection fraction was infrequently reported. Although heart failure with reduced ejection fraction was evenly distributed amongst the groups (see Tables 1 and 2), the reporting rate was in our opinion too low to include it into the ventricular events analysis. Furthermore, the use of propensity score matching, while mitigating some bias, cannot account for all potential confounders. The study was conducted at a single centre, which may limit the generalizability of the findings to other populations or clinical settings. Additionally, the relatively short follow-up might not have allowed the radiation therapy to reveal its full pro- or anti-arrhythmogenic effects.

The analysis relied on continuous rhythm monitoring from CIEDs, which, while comprehensive, does not reflect the arrhythmic burden in patients without such devices. Some devices did not have an atrial lead. Although it is one of the largest studies, the sample size is still relatively small and the event rate low, which limits the statistical power to detect differences and interactions between variables.

Future studies should aim to address these limitations through multi-centre, prospective cohort designs that include a broader patient population and more diverse clinical settings. Larger sample sizes will enhance the power to detect significant differences and interactions between risk factors.

Exploring the molecular and cellular changes in cardiac tissue post-radiotherapy, as previously done for single high dose irradiation, could elucidate the protective effects observed in this study [21]. In contrast to high-dose single radiation used as bail-out for severe non-controllable ventricular arrhythmia, the potentially arrhythmia-modulating effects of much lower and repetitive doses should be studied more in detail.

Given the significant role of beta-blockers in reducing arrhythmic burden during radiotherapy in our study, future research should focus on optimizing antiarrhythmic therapy protocols for patients undergoing radiotherapy. Randomized controlled trials could provide definitive evidence on the efficacy of beta-blockers, other antiarrhythmic agents or mineral corticoid receptor blockers, which are known to inhibit fibroblast activation, in this context.

In conclusion, this study highlights the complex relationship between radiotherapy and cardiac arrhythmias, revealing both risks and protective effects. The findings underscore the importance of personalized arrhythmia management strategies for patients undergoing radiotherapy, particularly those with a history of atrial fibrillation or coronary artery disease. Future research should build on these findings to develop targeted interventions that minimize the arrhythmic burden and improve the overall outcomes for cancer patients receiving radiotherapy.

## Supplementary Information

Supplementary Material 1.

## Data Availability

The anonymized data set used in this analysis will be shared upon reasonable request.
