# Supplementary material for: Cardiac arrhythmias during and after thoracic irradiation for malignancies
Source: Cardiooncology. 2024 Nov 14;10:81. doi: 10.1186/s40959-024-00277-3 (PMC11562486; doi:10.1186/s40959-024-00277-3)
Supplement: Supplementary file 1 — Supplementary Material 1. [file 40959_2024_277_MOESM1_ESM.docx]

**Supplementary Tables**

**Supplementary Table 1. Accuracy of automated full-text extraction.** 500 patients from the CIED patient cohort (10,026 and 49,377 reports) were randomly selected for manual confirmation of the automated extraction of information from full-text medical reports.

| **Variable** | **Extraction accuracy (%)** |
| --- | --- |
| Beta-blocker therapy | 99 |
| Arterial hypertension | 97 |
| Dyslipoproteinemia | 99 |
| Smoking status | 98 |
| Diabetes mellitus | 99 |
| Positive family history | 98 |
| Obesity | 100 |
| Coronary heart disease | 98 |
| Dilated cardiomyopathy | 100 |
| Hypertrophic cardiomyopathy | 99 |
| Atrial fibrillation | 96 |
| Sick sinus syndrome | 100 |
| Atrioventricular block | 97 |
| Atrial pacing | 94 |
| Ventricular pacing | 95 |
| Mode-switch time | 99 |
| AT/AF episodes | 97 |
| Non-sustained ventricular tachycardia | 97 |
| Sustained ventricular tachycardia | 99 |
| Ventricular fibrillation | 99 |
